# Immunosuppression at ICU admission is not associated with a higher incidence of ICU-acquired bacterial bloodstream infections: the COCONUT study

**DOI:** 10.1186/s13613-024-01314-1

**Published:** 2024-06-05

**Authors:** Ghadi Zebian, Louis Kreitmann, Marion Houard, Antoine Piantoni, Gaetan Piga, Sarah Ruffier des Aimes, Bérénice Holik, Frédéric Wallet, Julien Labreuche, Saad Nseir

**Affiliations:** 1 Médecine Intensive-Réanimation, Hôpital R. Salengro, CHU de Lille, Rue E. Laine, 59037 Lille Cedex, France; 2 Department of Intensive Care Medicine, Imperial College Healthcare NHS Trust, London, UK; 3 Centre for Antimicrobial Optimisation, Department of Infectious Disease, Faculty of Medicine, Imperial College London, London, W12 0HS UK; 4 Laboratoire de Bactériologie-Hygiène, CHU de Lille, Centre de Biologie Pathologie, 59000 Lille, France; 5 Department of Biostatistics, CHU Lille, 59000 Lille, France; 6 Inserm U1285, Université de Lille, CNRS, UMR 8576-UGSF, 59000 Lille, France

**Keywords:** Intensive care units, Critical illness, Immunocompromised hosts, Neoplasms, Bloodstream infections, Bacteremia, Cross infections, Antimicrobial resistance

## Abstract

**Background:**

Immunosuppression at intensive care unit (ICU) admission has been associated with a higher incidence of ICU-acquired infections, some of them related to opportunistic pathogens. However, the association of immunosuppression with the incidence, microbiology and outcomes of ICU-acquired bacterial bloodstream infections (BSI) has not been thoroughly investigated.

**Methods:**

Retrospective single-centered cohort study in France. All adult patients hospitalized in the ICU of Lille University-affiliated hospital for > 48 h between January 1st and December 31st, 2020, were included, regardless of their immune status. Immunosuppression was defined as active cancer or hematologic malignancy, neutropenia, hematopoietic stem cell and solid organ transplants, use of steroids or immunosuppressive drugs, human immunodeficiency virus infection and genetic immune deficiency. The primary objective was to compare the 28-day cumulative incidence of ICU-acquired bacterial BSI between immunocompromised and non-immunocompromised patients. Secondary objectives were to assess the microbiology and outcomes of ICU-acquired bacterial BSI in the two groups.

**Results:**

A total of 1313 patients (66.9% males, median age 62 years) were included. Among them, 271 (20.6%) were immunocompromised at ICU admission. Severity scores at admission, the use of invasive devices and antibiotic exposure during ICU stay were comparable between groups. Both prior to and after adjustment for pre‐specified baseline confounders, the 28-day cumulative incidence of ICU-acquired bacterial BSI was not statistically different between immunocompromised and non-immunocompromised patients. The distribution of bacteria was comparable between groups, with a majority of Gram-negative bacilli (~ 64.1%). The proportion of multidrug-resistant bacteria was also similar between groups. Occurrence of ICU-acquired bacterial BSI was associated with a longer ICU length-of-stay and a longer duration of invasive mechanical ventilation, with no significant association with mortality. Immune status did not modify the association between occurrence of ICU-acquired bacterial BSI and these outcomes.

**Conclusion:**

The 28-day cumulative incidence of ICU-acquired bacterial BSI was not statistically different between patients with and without immunosuppression at ICU admission.

**Supplementary Information:**

The online version contains supplementary material available at 10.1186/s13613-024-01314-1.

## Background

Critically ill patients are at increased risk for intensive care unit (ICU)-acquired infections [1] because of the frequent disruption of anatomic barriers by invasive devices (intravascular catheters, endotracheal tubes, etc.) and impairments in immune defenses present either at admission or acquired during ICU stay [2]. ICU-acquired bloodstream infections (BSI) are among the most prevalent ICU-acquired infections and are predominantly related to bacterial pathogens [3]. They are often classified as primary or secondary, the latter referring to cases where bacteremia occurs in the setting of a primary source of infection, most often hospital- and ventilator-associated pneumonia (HAP and VAP, respectively) and intravascular catheter-related BSI (CRBSI). Occurrence of ICU-acquired BSI is associated with a longer ICU length-of-stay and a higher mortality [4, 5], especially when antimicrobial treatment is postponed or inappropriate [6], or in the absence of appropriate source control [7, 8]. Initial empirical antibiotic treatment is challenging due to the rising prevalence of multidrug-resistant (MDR) bacteria in ICUs [9].

Immunocompromised patients account for an increasing proportion of users of the healthcare system as a result of recent advances in the treatment of cancer, hematologic malignancies and immune-mediated diseases [10]. In the last two decades, the mortality of immunocompromised patients hospitalized in ICUs has decreased substantially [11], and consequently their proportion in the typical ICU case-mix has increased to reach approximately a third of all ICU patients [12, 13]. There is clear evidence of an increased risk of community-acquired infections related to common and opportunistic pathogens in immunocompromised patients, especially those with neutropenia or hematologic malignancies [14–16]. While several recent studies have investigated the epidemiology of hospital- and ICU-acquired BSI [1, 3, 5, 7, 8, 17, 18], there is a paucity of data related specifically to immunocompromised patients. Immunosuppression is often cited as a risk factor for ICU-acquired BSI [3], but has not been confirmed in several recent studies [4, 5, 18], and the association of immune status with the microbiology and outcomes of BSI is unclear.

To investigate this, we conducted the COCONUT study, a retrospective single-center study in the ICU of Lille University-affiliated hospital. The primary objective was to examine the association between immunosuppression at ICU admission and the 28-day cumulative incidence of ICU-acquired bacterial BSI. We reasoned that immunocompromised patients are often exposed to several risk factors for ICU-acquired BSI (including long-term vascular catheters such as implanted ports or peripherally implanted central catheters), thus our hypothesis was that the incidence of ICU-acquired bacterial BSI would be higher in immunocompromised than in non-immunocompromised patients. Secondary objectives included: (1) to describe the microbiology of ICU-acquired bacterial BSI in immunocompromised and non-immunocompromised patients; (2) to examine the association between occurrence of BSI and patient outcomes; (3) to assess whether immune status modifies the association between occurrence of BSI and patient outcomes; and (4) to examine the association between immunosuppression at ICU admission and patient outcomes.

## Methods

### Population and definitions

The COCONUT study (ICU-acquired blOodstream infeCtiONs in immUnocompromised paTients) was a retrospective single-center observational study at the ICU of Lille University-hospital (France). All adult patients hospitalized for > 48 h between January 1st and December 31st, 2020 were included, regardless of their immune status.

Immunosuppression was defined as solid cancer or hematologic malignancy (active or in remission for less than 5 years), neutropenia (neutrophil count < 1.5 G/L), hematopoietic stem cell transplant (HSCT), solid-organ transplant, long-term (≥ 28 days) use of steroids (at a dose ≥ 10 mg of prednisone per day or equivalent) or other immunosuppressant drugs, human immunodeficiency virus (HIV) infection, and genetic immune deficiency [13]. Immunological studies were not performed to further characterize immune functions among patients recruited to the study.

### Data collection

Data were extracted from healthcare records into an electronic case report form. Data collected at baseline included: age, gender, body mass index (BMI), dates of ICU admission and discharge, Simplified Acute Physiology Score (SAPS) II score [19], Sequential Organ Failure Assessment(SOFA) [20], immune status at ICU admission, comorbidities, recent (i.e., in the 3 months before ICU admission) hospitalization for > 48 h, recent surgery, recent antibiotic treatment or known colonization with MDR bacteria, type of admission (medical vs. surgical), COVID-19 status, location before ICU admission, and reason for ICU admission.

Data collected during ICU stay included: invasive devices (central venous, arterial, dialysis catheters, endotracheal tube and tracheostomy), duration of invasive mechanical ventilation (IMV), prone positioning, extracorporeal membrane oxygenation (ECMO) or extracorporeal life support (ECLS), treatments received during ICU stay (including parenteral nutrition, transfusion, antibiotics and steroids), ICU-acquired colonization with MDR bacteria and ICU-acquired BSI.

### Infection control and prevention

All patients enrolled in the study were hospitalized in single-bed ICU rooms. Infection control and prevention (IPC) measures routinely used in our center are in line with European guidelines, including contact precautions and isolation measures (as indicated), specifically with enhanced air filtration and positive room air pressure for high risk patients, and the prompt removal of catheters for all patients. Chlorhexidine bathing and selective digestive decontamination are not used routinely.

### Endpoints

The primary endpoint was the 28-day cumulative incidence of ICU-acquired bacterial BSI, and was compared between immunocompromised and non-immunocompromised patients. ICU-acquired BSI related to fungi were not considered. Secondary endpoints included all-cause ICU mortality, ICU length-of-stay and duration of IMV, all censored at day 28 post-ICU admission.

### Microbiology

The diagnosis of ICU-acquired bacterial BSI was based on a positive blood culture in the context of hyperthermia (temperature > 38 °C) or elevated blood markers of inflammation (C-reactive protein [CRP] or procalcitonin) occurring at least 48 h after ICU admission [21]. A single positive blood culture was sufficient to diagnose ICU-acquired BSI for most bacteria except for skin commensals (coagulase-negative staphylococci [CNS]*, Bacillus* spp.*, Corynebacterium* spp., and *Cutibacterium acnes*). In those cases, at least two sets of positive blood cultures collected from different sites or at a different time points were needed to rule out contamination. The time between sampling of different sets of blood cultures was not taken into consideration [22]. Blood cultures positive with fungi were excluded. ICU-acquired BSI was deemed secondary to another infection in cases where patients fulfilled criteria for another infection—including HAP, VAP [23] and CRBSI [21]—with the same microorganisms at the time blood cultures were sampled. Only the first episode of ICU-acquired BSI was considered.

Bacteria were identified by matrix-assisted laser desorption ionization time of flight mass spectrometry (MALDI-TOF-MS) with a Microflex mass spectrometer (Bruker Daltonik S.A., Wissembourg, France) according to the manufacturer’s instructions after extraction using formic acid. Antimicrobial susceptibility testing was performed using the Vitek-2 system (bioMérieux, Marcy-l’Étoile, France), combined with the MASTDISCS ID ESBL detection disc diffusion tests (Mast Diagnostics, Amiens, France) to confirm the presence of an extended spectrum beta-lactamase (ESBL) or the overexpression of a cephalosporinase. In case of carbapenemase, the OKNVI Resist Coris test (CorisBioconcept, Gembloux, Belgium) was used to determine the type of carbapenemase. Clinical breakpoints were interpreted using criteria proposed by the *Comité de l’Antibiogramme de la Société Française de Microbiologie* (CA-SFM EUCAST 2019) [24]. MDR bacteria were defined as: third generation cephalosporins (3GC)-resistant *Enterobacteriaceae*, including through expression of an ESBL; carbapenem-resistant *Enterobacteriaceae*; methicillin-resistant *Staphylococcus aureus* (MRSA); vancomycin-resistant *Enterococcus faecalis* and *Enterococcus faecium* (VRE); *Pseudomonas aeruginosa* resistant to imipenem and ceftazidime; and carbapenem-resistant *Acinetobacter baumannii* (CRAB) [25].

### Statistical analysis

Patient characteristics at ICU admission and during ICU stay were described according to immune status without statistical comparisons. Categorical variables were reported as number and percentage, whereas quantitative variables were expressed as median (and 25th to 75th percentiles).

We assessed the risk of ICU-acquired bacterial BSI using competing risk survival analysis to take into account the duration of ICU stay by treating ICU discharge (alive or dead) as a competing event. We also used competing risk survival analysis to analyze ICU mortality (considering ‘death in ICU’ as event of interest and ‘ICU discharge alive’ as competing event), duration of IMV (considering ‘successful weaning’ as event of interest and ‘death under IMV’ as competing event) and ICU length-of-stay (considering ‘ICU discharge alive’ as event of interest and ‘death in the ICU’ as competing event). In survival analyses, start time was set at the date of ICU admission for ICU mortality and ICU length-of-stay, and as the date of first intubation for duration of IMV (analysis carried out in a subset of 666 patients under IMV). All analyses were censored at 28 days.

We estimated the cumulative incidence of ICU-acquired bacterial BSI, ICU mortality, successful weaning of IMV and ICU discharge alive according to immune status by using the non-parametric Kalbfleisch and Prentice method to account for competing events [26]. The association of immune status with each outcomes was assessed using cause-specific Cox proportional hazard models regarding the causal research question [27]. We chose to use cause-specific Cox models rather than Fine and Gray models because our aim was to assess etiological associations [27, 28]. Cause-specific hazard ratios (cHR) for immunocompromised vs. non-immunocompromised were derived from Cox regression models with theirs 95% confidence intervals (CI) as effect size, and the proportional hazard assumption was assessed by using the scaled Schoenfeld residuals plots. The association of immune status with the risk of ICU-acquired bacterial BSI was further investigated after adjustment for pre-specified baseline confounders (age, gender, COVID-19, SAPS-II and SOFA scores) and pre-specified time-varying confounders (exposure to central venous catheters, arterial catheters, renal replacement therapy, IMV and antibiotic treatment in ICU). The association of immune status with prognostic outcomes (ICU mortality, duration of IMV and ICU length-of-stay) was further investigated after adjustment for pre-specified baseline confounders (age, gender, COVID-19, SAPS-II, heart failure, chronic respiratory disease, chronic kidney disease). To account for the fact that the proportional hazard assumption was violated for COVID-19 in all Cox models, the effect of COVID-19 status was modeled by including time-dependent coefficients in the multivariable Cox models. For the duration of IMV and ICU length-of-stay, the proportional hazard assumption for SAPS-II was also not satisfied, thus the effect of SAPS-II was also modeled by including time-dependent coefficients. As a secondary analysis, we also estimated and compared the incidence rates of ICU-acquired bacterial BSI (expressed as number of events per 1000 ICU days, and per 1000 catheter days for patients with at least one ICU day with catheter) of ICU-acquired bacterial BSI according to immune status by using a Poisson regression model, using ICU duration (or catheter duration) as offset variable (after applying a log-transformation), before and after adjustment for pre-specified baseline confounders.

We investigated the association of occurrence of ICU-acquired bacterial BSI with prognostic outcomes by using univariable and multivariable cause-specific Cox regression models, treating ICU-acquired BSI as a time-varying covariate. The same confounders included in analyses of the association of immune status with prognostic outcomes were included in these models. In addition, we did a subgroup analysis of the association of ICU-acquired bacterial BSI and patient prognostic outcomes according to immune status by fitting separate cause-specific Cox regressions models. Heterogeneity in the association of occurrence of ICU-acquired BSI with patient outcomes according to immune status was assessed using the chi-square heterogeneity test.

Statistical testing was performed with a two-tailed α level of 0.05. Data were analyzed using the SAS software package, release 9.4 (SAS Institute, Cary, NC).

## Results

### Patients characteristics

A total of 1313 patients were included between January 1st and December 31st, 2020. Among them, 271 (20.6%) were immunocompromised and 1042 (79.4%) were non-immunocompromised at ICU admission. The main causes of immunosuppression were the use of immunosuppressive therapies (n = 134, 49.4%), cancer (n = 103, 38.0%), steroids (n = 90, 33.2%), hematologic malignancy (n = 78, 28.8%) and neutropenia (n = 48, 17.7%). One hundred and seventy patients (62.7%) had more than one cause of immunosuppression (Supplementary Table 1).

Patients were mostly male (66.9%), with a median age of 62 years (Table 1). Some comorbidities were more common among immunocompromised patients, including chronic cardiovascular disease, chronic lung disease and chronic kidney disease. Immunocompromised patients were more likely than non-immunocompromised patients to have been hospitalized on a ward for > 48 h, to have had surgery and to have received antibiotics for > 48 h in the 3 months prior to ICU admission. The proportion of patients colonized with MDR bacteria at ICU admission was also higher among immunocompromised patients than among non-immunocompromised patients. The type of ICU admission, severity scores, exposure to invasive devices, use and duration of antibiotics during ICU stay were comparable between groups. Transfusion of blood products and corticosteroids exposure during ICU stay were more frequent in immunocompromised patients than in non-immunocompromised patients. However, the doses of steroids received during ICU stay were similar between groups (Table 2).

**Table 1:** Patient characteristics at ICU admission

Characteristics	Overall cohort (n = 1313)	Immuno-compromised patients (n = 271)	Non-immuno-compromised patients (n = 1042)
Age (years)	62 (50–70)	65 (54–72)	61 (50–70)
Male gender	8793 (66.9)	165 (60.9)	714 (68.5)
Body mass index (kg/m^2^)	27.4 (23.7–32.7)^1^	25.6 (22.5–30.1)^2^	27.9 (24.0–33.7)^3^
Smoking	366 (27.9)	72 (26.6)	294 (28.2)
Chronic alcohol use	216 (16.5)	25 (9.2)	191 (18.3)
Diabetes mellitus	390 (29.7)	71 (26.2)	319 (30.6)
Cardiovascular disease	719 (54.8)	164 (60.5)	555 (53.3)
Hypertension	642 (48.9)	143 (52.8)	499 (47.9)
Coronary-artery disease	188 (14.3)	39 (14.4)	149 (14.3)
Heart failure	154 (11.7)	42 (15.5)	112 (10.7)
Venous thromboembolic disease	86 (6.5)	28 (10.3)	58 (5.6)
Lung disease	284 (21.6)	76 (28.0)	208 (20.0)
Chronic respiratory disease	250 (19.0)	70 (25.8)	180 (17.3)
COPD	177 (13.5)	37 (13.7)	140 (13.4)
Chronic kidney disease	128 (9.7)	40 (14.8)	88 (8.4)
Liver cirrhosis	56 (4.3)	16 (5.9)	40 (3.8)
Recent surgery	111 (8.5)	39 (14.4)	72 (6.9)
Recent antibiotic treatment for > 48 h	327 (24.9)	107 (39.5)	220 (21.1)
Recent hospitalization for > 48 h	351 (26.7)	132 (48.7)	219 (21.0)
Known colonization with MDR bacteria	149 (11.3)	48 (17.7)	101 (9.7)
COVID-19	488 (37.2)	64 (23.6)	424 (40.7)
SAPS-II	39 (29–55)^4^	44 (34–57)	38 (28–54)^5^
SOFA score	4 (2–8)^6^	4 (2–7)^7^	4 (2–8)^8^
Type of ICU admission			
Medical	1228 (93.5)	256 (94.5)	972 (93.5)
Surgical	853 (6.5)	15 (5.5)	70 (6.7)

Values are as number (%), or median (25th to 75th percentiles)

*COPD* chronic obstructive pulmonary disease, *ICU* intensive care unit, *MDR* multidrug-resistant, *SAPS-II* simplified acute physiology score II, *SOFA* sequential organ failure assessment

Missing values: ^1^111, ^2^15, ^3^96, ^4^2, ^5^2, ^6^80, ^7^19, ^8^61

**Table 2:** Patient characteristics during ICU stay

Characteristics	Overall cohort (n = 1313)	Immuno-compromised patients (n = 271)	Non-immuno-compromised patients (n = 1042)
Invasive devices and procedures			
Central venous catheter	804 (61.2)	177 (65.3)	627 (60.2)
Duration (days)	10 (6–20)	9 (5–20)	11 (6–22)
Arterial catheter	944 (71.9)	198 (73.1)	746 (71.6)
Duration (days)	10 (6–18)	9 (5–14)	10 (6–20)
Renal replacement therapy	137 (10.4)	24 (8.9)	113 (10.8)
Invasive mechanical ventilation	666 (50.7)	122 (45.0)	544 (52.2)
Duration (days)	8 (3–18)	7 (3–14)	9 (3–19)
Treatments			
Antibiotics	1127 (85.8)	237 (87.5)	890 (85.4)
Duration (days)	5 (3–7)^1^	5 (3–8)^2^	5 (3–7)^3^
Parenteral nutrition	111 (8.5)	18 (6.6)	93 (8.9)
Transfusions	326 (24.8)	93 (34.3)	233 (22.4)
Corticosteroids	615 (46.8)	153 (56.4)	462 (44.3)
Prednisone dose equivalence (mg)	60 (40–130)^4^	50 (40–100)^5^	62 (40–130)^6^

Values are as number (%) or median (25th to 75th percentiles)

*ICU* intensive care unit

Missing values: ^1^9, ^2^1, ^3^8, ^4^31, ^5^9, ^6^22

### Association between immune status and the incidence of ICU-acquired bacterial BSI

**Fig. 1:**
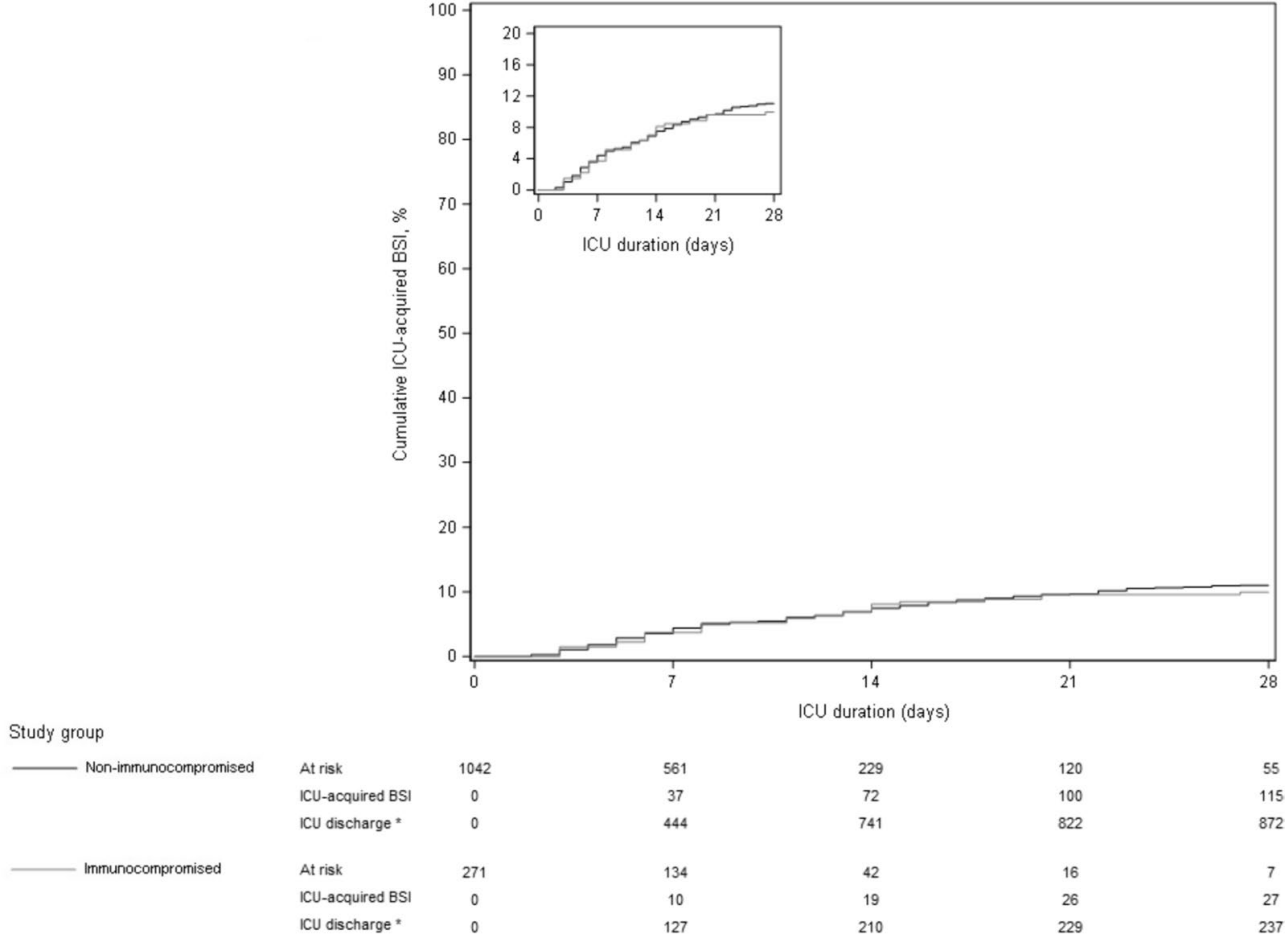
Cumulative incidence of ICU-acquired BSI according to immune status, considering death as a competing event

As shown in Fig. 1 and Table 3, 27 immunocompromised patients over 271 (incidence, 10.0%) presented at least one episode of ICU-acquired bacterial BSI in the 28 days following ICU admission, in comparison to 115 over 1042 non-immunocompromised patients (incidence, 11.0%). In cause-specific Cox regression analysis, the occurrence of ICU-acquired bacterial BSI was not associated with immune status, both in univariate analysis (cHR 1.12, 95% CI 0.73–1.70) and after adjustment for pre-specified confounders (adjusted cHR 1.57, 95% CI 0.99–2.49).

**Table 3:** Association of immunosuppression at ICU admission with ICU-acquired BSI and main prognostic outcomes

28-day outcomes	Non-immuno-compromised patients (n = 1042)	Immuno-compromised patients (n = 271)	Unadjusted		Adjusted	
			cHR (95% CI)	*P*-Value	cHR (95% CI)	*P*-Value
ICU-acquired BSI^1^						
Cumulative incidence (%)	115 (11.0)	27 (10.0)	1.12 (0.73–1.70)	0.60	1.57 (0.99–2.49)^2^	0.053
Incidence rate (95%CI) per 1000 ICU-days	20.8 (18.3–23.7)	22.9 (17.6–30.0)	1.10 (0.82–1.47)^3^	0.52	1.33 (0.97–1.80)^3,4^	0.069
Incidence rate (95%CI) per 1000 catheter-days^5^	25.3 (22.2–28.9)	29.3 (22.6–37.9)	1.16 (0.86–1.55)^3^	0.32	1.38 (1.01–1.89)^3,4^	0.038
28-day mortality	166 (15.9)	79 (29.2)	2.10 (1.60–2.75)	< 0.001	1.81 (1.37–2.41)^6^	< 0.001
ICU discharge alive	777 (74.6)	176 (64.9)	0.97 (0.82–1.15)	0.75	0.88 (0.74–1.04)^6^	0.13
Successful weaning of IMV	408 (75.9)	69 (56.6)	0.82 (0.63–1.07)	0.14	0.68 (0.52–0.89)^6^	0.005

Values are number of events (28-day cumulative incidence, in %) otherwise as indicated. IMV analysis was done in the 666 patients treated by IMV during the first 28 days of ICU stay

*BSI* bloodstream infections, *CI* confidence interval, *cHR* cause-specific hazard ratio, *IMV* invasive mechanical ventilation, *ICU* intensive care unit, *SAPS-II* simplified acute physiology Score II, *SOFA* sequential organ failure assessment

^1^Pre-specified as primary outcome

^2^Adjusted for pre-specified baseline confounders (age, gender, COVID-19**,** SAPS-II and SOFA scores) and pre-specified time-dependent confounders (exposure to central venous catheters, arterial catheters, renal replacement therapy, IMV and antibiotic treatment in ICU)

^3^Incidence rate ratio

^4^Adjusted for pre-specified baseline confounders (age, gender, COVID-19**,** SAPS-II and SOFA scores)

^5^Calculated in 956 patients with a catheter for at least one day

^6^Adjusted for pre-specified baseline confounders (age, gender, COVID-19, SAPS-II, heart failure, chronic respiratory disease, chronic kidney disease)

### Microbiology

Among the bacteria responsible for ICU-acquired BSI, Gram-negative bacilli were the most frequent organisms identified (64.1%), mainly *Klebsiella pneumoniae* and *Enterobacter* spp*.*, followed by Gram-positive cocci (34.5%), mainly coagulase-negative staphylococci (Supplementary Table 2). The distribution of bacteria was comparable between groups. The distribution of BSI sources was also comparable between groups, with a majority of secondary BSI related to VAP, followed by CRBSI.

We identified a total of 47 ICU-acquired BSIs related to MDR bacteria (33.1%). The proportion of ICU-acquired BSI related to MDR bacteria was comparable between groups (29.6% in immunocompromised vs. 33.9% in non-immunocompromised patients). Among those MDR bacteria, 3GC-resistant *Enterobacteriaceae* were the most frequently isolated organisms (63.8%), followed by carbapenem-resistant *Enterobacteriaceae*, imipenem-resistant *Acinetobacter* spp. and MRSA (Supplementary Table 2).

### Association between ICU-acquired bacterial BSI and prognosis

**Fig. 2:**
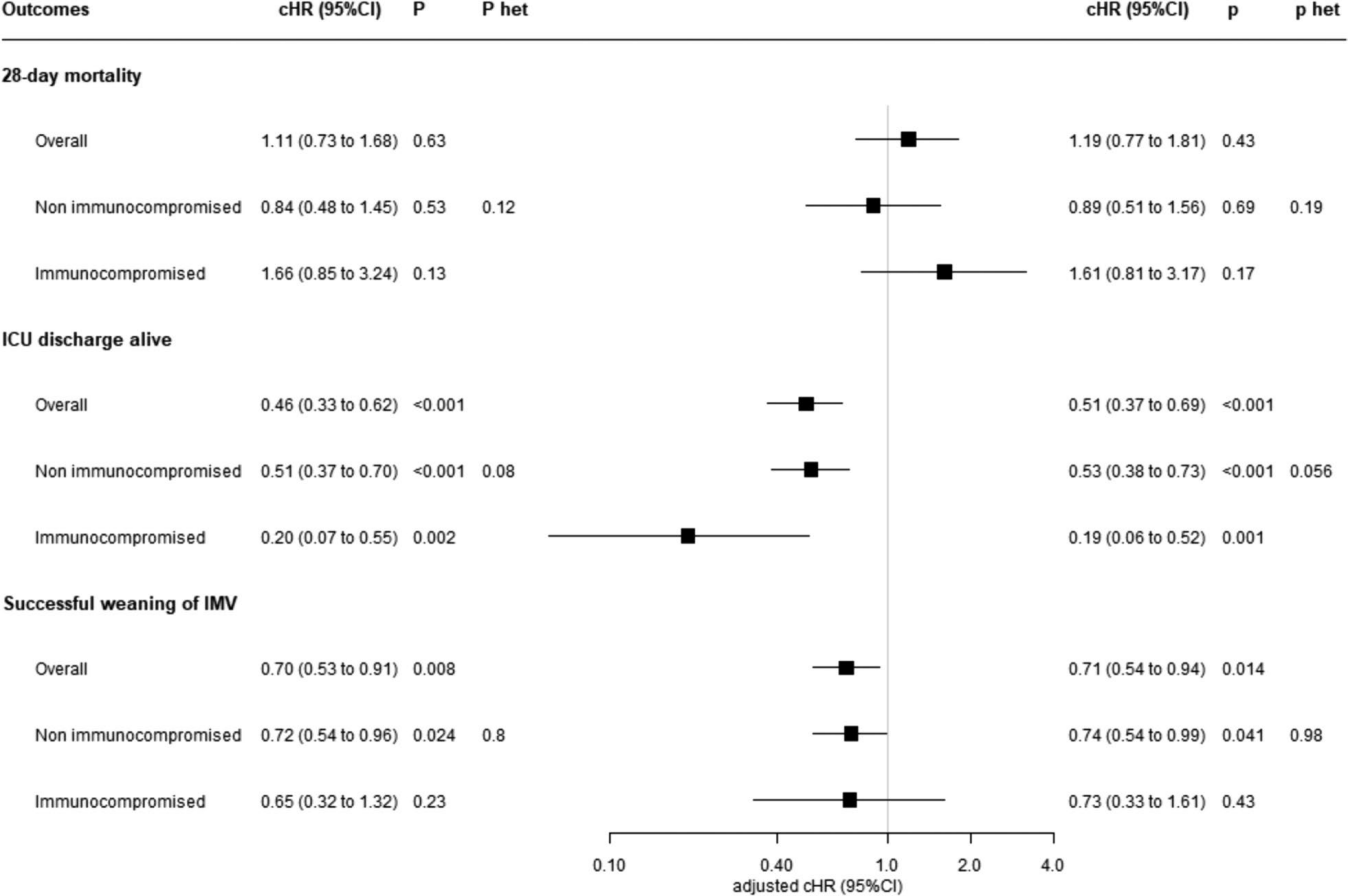
Unadjusted and adjusted effect size for occurrence of ICU-acquired BSI on ICU mortality, ICU discharge alive and successful weaning of IMV (censored at day 28). cHRs were calculated using Cox proportional hazard models with a cause-specific hazard approach, by treating ICU-acquired BSI as a time-dependent binary covariate, with adjustment on pre-specified baseline confounders (age, gender, COVID-19, SAPS-II, heart failure, chronic respiratory disease, chronic kidney disease). P-het indicates p-values for heterogeneity (i.e., p-value for comparison in effect size associated with ICU-acquired BSI between non-immunocompromised and immunocompromised patients). A cHR > 1 indicates a decrease in ICU survival (i.e., an increased risk of mortality), duration of IMV (i.e., an increased risk of successful weaning) and ICU length-of-stay (i.e., an increased risk of ICU discharge alive). Conversely, a cHR < 1 indicates an increase in ICU survival (i.e., a decreased risk of mortality), duration of IMV (i.e., a decreased risk of successful weaning) and ICU length-of-stay (i.e., a decreased risk of ICU discharge alive). Note that the event of interest for ICU survival is a pejorative event (death), whereas for duration of IMV and ICU length-of-stay the event of interest is a positive event (successful weaning or ICU discharge alive). *BSI* bloodstream infections, *CI* confidence interval, *cHR* cause-specific hazard ratio, *ICU* intensive care unit, *IMV* invasive mechanical ventilation, *SAPS-II* simplified acute physiology Score II

Considering the whole cohort, there was no significant association between occurrence of ICU-acquired bacterial BSI and ICU mortality during the first 28 days following ICU admission (adjusted HR 1.19, 95% CI 0.77–1.81) (Fig. 2). Occurrence of ICU-acquired bacterial BSI was associated with a longer ICU length-of-stay (adjusted cHR 0.51, 95% CI 0.37–0.69 for the event ‘ICU discharge alive’) and a longer duration of IMV (adjusted cHR 0.71, 95% CI 0.54–0.94 for the event ‘successful weaning of IMV’). There was no evidence of heterogeneity in the association between occurrence of ICU-acquired bacterial BSI and prognostic outcomes according to immune status, i.e., comparable associations were found when considering immunocompromised and non-immunocompromised patients separately.

### Association between immune status and prognosis

As shown in Table 3 and Supplementary Fig. 1, immunosuppression was associated with a higher 28-day ICU mortality (adjusted cHR 1.81, 95% CI 1.37–2.41) and a longer duration of IMV (among the 666 patients receiving IMV, adjusted cHR 0.68, 95% CI 0.52–0.89 for the event ‘successful weaning of IMV’). No significant association was found between immune status and ICU length-of-stay.

## Discussion

In this observational retrospective single-center cohort study, we found that the incidence of ICU-acquired bacterial BSI was not significantly different between immunocompromised and non-immunocompromised patients. The microbiology of BSI was similar between groups. The occurrence of ICU-acquired bacterial BSI was associated with longer ICU length-of-stay and duration of IMV, but not with an increased mortality.

There is a wealth of epidemiological evidence demonstrating that immunocompromised patients are at higher risk than non-immunocompromised patients for infections in general [14, 15], and for community-acquired infections in particular, including community-acquired BSI [16] and pneumonia [29]. It is also clear that patients with profound immunosuppression, especially patients with prolonged neutropenia or hematologic malignancies, are at higher risk of hospital-acquired infections, and that these infections carry a higher risk of worse outcomes in this population. However, few studies have specifically investigated the association between immunosuppression—using a broader definition including patients with different types of immunosuppression—and ICU-acquired infections, especially ICU-acquired BSI. Interestingly, immunosuppression of any cause at ICU admission was not a risk factor for the occurrence of ICU-acquired BSI in a retrospective analysis on 571 ICU-acquired BSI episodes among 10,734 patients from the Outcomerea Database (France) [4], nor in a retrospective study on 1306 ICU-acquired BSI episodes among 150,948 ICU admissions in 85 American ICUs [18]. In a retrospective study on 330 ICU-acquired BSI episodes among 6339 patients in Australia, immune deficiency and malignancies were more prevalent in patients with at least one ICU-acquired BSI than in patients without (10.6 vs. 7.0%, p = 0.02 for immunosuppression and 19.1 vs. 14.8%, p = 0.04 for malignancies), but immunosuppression was not an independent risk factor for ICU-acquired BSI in multivariate analysis [5]. Overall, the practical implication of these findings—if they are confirmed in subsequent larger multicenter studies—is that the level of clinical suspicion, the microbiological work-up and the management of ICU-acquired bacterial BSI should not differ between immunocompromised and non-immunocompromised patients.

Several factors could explain these somewhat counter-intuitive findings. First among these is antibiotic exposure in the ICU, which can modulate the risk of ICU-acquired BSI in several ways. On the one hand, exposure to antibiotics (especially if broad-spectrum) could lead to a decreased sensitivity of blood cultures, and consequently induce a bias in our results towards a lower rate of detection of BSI. On the other hand, antibiotics also have an untargeted effect on the normal commensal flora, including that of the skin, which leads to a decreased resistance to colonization by pathogenic strains and can facilitate secondary infections [30]. In the COCONUT study, we did not record antibiotic exposure with enough granularity to characterize this further. However, the number of days on antibiotics was similar in immunocompromised and non-immunocompromised patients, and this variable was accounted for by multivariate analysis when assessing the association of immune status with the incidence of ICU-acquired BSI.

Second, the sources of ICU-acquired bacterial BSI should be considered. In the COCONUT study, the primary sources of ICU-acquired BSI were pulmonary infections, including VAP. Contrary to common assumptions, we have shown in an ancillary analysis of the prospective multinational TAVeM database that the incidence of ventilator-associated lower respiratory tract infection was lower in immunocompromised than non-immunocompromised patients (16.6% vs. 24.2%, respectively, subdistribution HR 0.65, 95% CI 0.53–0.80) [31]. This could explain in part why, in the COCONUT study, the incidence of ICU-acquired bacterial BSI was not higher in immunocompromised than in non-immunocompromised patients.

Third, we adopted a broad definition of immunosuppression, and it is probable that our findings also reflect a substantial heterogeneity in the nature, depth and duration of immune defects in this patient group. A detailed analysis of the risk of ICU-acquired BSI in patients with different types of immunosuppression (e.g., neutropenic patients vs. others) could provide more detailed insight into this, but our limited sample size precluded such analysis in this cohort. Furthermore, it is now well established that a large proportion of patients with an apparently normal immune system at baseline develop features of acquired immunosuppression as a result of the initial insult—sepsis, major surgery or trauma—that precipitated their ICU admission, or because they are exposed to immune-modulating therapies in the ICU [32, 33]. This might explain that the ‘actual net state of immunosuppression’—an ill-defined concept that is for now impossible to quantify precisely at the bedside—could actually be comparable between patients labeled as ‘immunocompromised’ at admission and their apparently non-immunocompromised counterparts.

In the COCONUT study, there was no significant difference in the proportion of ICU-acquired BSI related to MDR bacteria among immunocompromised and non-immunocompromised patients. These findings are in line with a recent observational multicenter study where the incidence of ICU-acquired infections with MDR bacteria (including BSI) were not different between these two patient groups [13]. Whether this relates to differences in IPC strategies or other factors (differential exposure to antimicrobials, immune functions) remains to be explored specifically.

We found that the occurrence of ICU-acquired bacterial BSI was associated with longer ICU length-of-stay and duration of IMV, but was not significantly associated with mortality. This is in contradiction with several studies which have documented an association between occurrence of ICU-acquired BSI and higher mortality, including the study by Adrie et al. (adjusted HR 1.40, 95% CI 1.16–1.69) [4], the study by Prowle et al. (adjusted HR 2.89, 95% CI 2.41–3.46) [5], and a retrospective study on 232 ICU-acquired BSI episodes among 3247 patients in 12 ICUs in France (odds ratio [OR] 3.20 95% CI 2.30–4.43) [6]. This could be explained by a lack of statistical power to detect an impact on mortality, due to a smaller sample size (and subsequently a small number of ICU-acquired BSI) in the COCONUT study.

Little data has been published on the way baseline immunosuppression modifies the association between occurrence of ICU-acquired BSI and outcomes. In the EUROBACT study, Tabah et al. found that among patients with hospital-acquired BSI (76% of which were acquired in the ICU), immunosuppression was associated with an increased mortality risk (OR 2.11, 95% CI 1.40–3.19) [7]. However, in the COCONUT study we found that immunosuppression at ICU admission had no effect on the association between occurrence of BSI and patient outcomes. This could be due to different definitions of immunosuppression, to the evolution of practices between these two studies, or to a lack of power in the COCONUT study. Several studies have documented a clear positive impact of source control to reduce mortality related to BSI [7, 8], but unfortunately the proportion of patients achieving prompt source control in our cohort was not recorded. However, because it is standard practice at our institution to promptly change all central venous and arterial lines in case of ICU-acquired sepsis of unknown origin—independently of immune status—this could also explain why mortality associated with the occurrence with BSI was similar in immunocompromised and non-immunocompromised patients.

Our study has several limitations. Its retrospective and mono-centric design make it difficult to extend our findings to other ICU settings. Due to its limited sample size and the relatively low incidence of ICU-acquired bacterial BSI, it is possible that a small but substantial difference in the incidence of ICU-acquired BSI between immunocompromised and non-immunocompromised patients could not be detected because of a lack of statistical power. This is also suggested by the fact that the adjusted cause-specific hazard ratio for the incidence of ICU-acquired BSI almost reaches statistical significance (indicating a higher risk in immunocompromised patients) after adjustment for confounders. We did not record detailed data on antibiotic use, a key determinant of the epidemiology of ICU-acquired infections in general, and ICU-acquired BSI in particular. We acknowledge that our definition of immunosuppression is imperfect, as it groups together patients with clearly heterogeneous immune dysfunctions, and fails to capture ICU-acquired immune defects known to be associated with the occurrence of ICU-acquired infections. The limited sample size precluded a more detailed analysis aiming to compare the incidence, microbiology and outcomes of ICU-acquired BSI between patients with different types of immunosuppression (e.g., neutropenic patients vs. others). Finally, we did not collect data on antibiotic therapy initiated after identification of ICU-acquired BSI episodes, and it would have been interesting to assess whether the appropriateness of initial empiric antibiotic treatments had an impact of the association between ICU-acquired BSI and outcomes.

## Conclusion

In this monocentric, retrospective observational cohort study, the incidence of ICU-acquired bacterial BSI was not different between immunocompromised and non-immunocompromised patients. This suggests that the clinical management of ICU-acquired bacterial BSI should not differ between immunocompromised and non-immunocompromised patients. Further studies are required to better assess the relationship between immunosuppression—both present at ICU admission or acquired during ICU stay—and the incidence, microbiology and outcomes of ICU-acquired infections in general, and ICU-acquired BSI specifically.

### Supplementary Information

Supplementary Material 1.

## Data Availability

The datasets used and/or analyzed during the current study are available from the corresponding author on reasonable request.

## Abbreviations

3GC: Third-generation cephalosporin
BMI: Body mass index
BSI: Bloodstream infection
COPD: Chronic obstructive pulmonary disease
CRBSI: Intravascular catheter-related bloodstream infection
ECLS: Extra-corporeal life support
ECMO: Extra-corporeal membrane oxygenation
ESBL: Extended spectrum beta-lactamase
HIV: Human immunodeficiency virus
HR: Hazard ratio
cHR: Cause-specific hazard ratio
HSCT: Hematopoietic stem cell transplant
ICU: Intensive care unit
IMV: Invasive mechanical ventilation
MDR: Multidrug resistant
MRSA: Methicillin-resistant *Staphylococcus aureus*
SAPS-II: Simplified Acute Physiology Score
SOFA: Sequential Organ Failure Assessment
VA-LRTI: Ventilator-associated lower respiratory tract infection
VAP: Ventilator-associated pneumonia
VAT: Ventilator-associated tracheobronchitis
VRE: Vancomycin-resistant Enterobacterales
