# Supplementary material for: Immunosuppression at ICU admission is not associated with a higher incidence of ICU-acquired bacterial bloodstream infections: the COCONUT study
Source: Ann Intensive Care. 2024 Jun 5;14:83. doi: 10.1186/s13613-024-01314-1 (PMC11153408; doi:10.1186/s13613-024-01314-1)

**Immunosuppression at ICU admission is not associated with a higher incidence**

**of ICU-acquired bloodstream infections: the COCONUT study**

**Supplementary material**

**Supplementary table 1. Causes of immunosuppression in the 249 patients with immunosuppression at ICU admission**

| Causes of immunosuppression | Overall  (n= 271) | No ICU-acquired BSI (n=244) | ICU-acquired BSI  (n=27) |
| --- | --- | --- | --- |
| Hematologic malignancy | 78 (28.8) | 71 (29.1) | 7 (25.9) |
| Neutropenia | 48 (17.7) | 39 (15.9) | 9 (33.3) |
| Hematopoietic stem cell transplant | 6 (2.2) | 4 (1.6) | 2 (7.4) |
| Solid organ transplant recipient | 33 (12.2) | 29 (11.9) | 4 (14.8) |
| Immunosuppressive drugs | 134 (49.4) | 124 (50.8) | 10 (37.0) |
| Steroids | 90 (33.2) | 77 (31.1) | 14 (51.9) |
| Cancer | 103 (38.0) | 98 (40.2) | 5 (18.5) |
| Metastatic cancer | 49 (18.1) | 48 (19.7) | 1 (3.7) |
| HIV | 2 (0.7) | 2 (0.8) | 0 (0.0) |
| Other | 5 (1.8) | 5 (2.0) | 0 (0.0) |
| Multiple causes of immunosuppression | 170 (62.7) | 156 (63.9) | 14 (51.9) |

Values are no. (%) unless otherwise indicated.

Abbreviations: BSI: bloodstream infection; ICU: intensive care unit; HIV: human immunodeficiency virus.

**Supplementary table 2. Microbiology of ICU-acquired BSI**

| Microbiology | Overall (n=142) | Immuno-compromised  patients  (n=27) | Non-immuno-compromised  patients  (n=115) |
| --- | --- | --- | --- |
| **Type of bacteria** |  |  |  |
| Gram positive cocci | 49 (34.5) | 5 (18.5) | 44 (38.3) |
| Coagulase negative staphylococci | 23 (16.2) | 0 (0.0) | 23 (20.0) |
| *Staphylococcus aureus* | 9 (6.3) | 2 (7.4) | 7 (6.1) |
| *Enterococcus* spp*.* | 15 (10.6) | 3 (11.1) | 12 (10.4) |
| *Streptococcus pneumoniae* | 0 (0.0) | 0 (0.0) | 0 (0.0) |
| Other Gram positive cocci | 2 (1.4) | 0 (0.0) | 2 (1.7) |
| Gram negative bacilli | 91 (64.1) | 20 (74.1) | 71 (61.7) |
| *Klebsiella pneumoniae* | 24 (16.9) | 7 (25.9) | 17 (14.8) |
| *Escherichia coli* | 9 (6.3) | 1 (3.7) | 8 (7.0) |
| *Enterobacter* spp*.* | 20 (14.1) | 2 (7.4) | 18 (15.7) |
| Other Enterobacteriaceae | 15 (10.6) | 5 (18.5) | 10 (8.7) |
| *Pseudomonas aeruginosa* | 14 (9.9) | 5 (18.5) | 9 (7.8) |
| *Acinetobacter baumannii* | 7 (4.9) | 0 (0.0) | 7 (6.1) |
| *Stenotrophomonas maltophilia* | 2 (1.4) | 0 (0.0) | 2 (1.7) |
| Anaerobes | 2 (1.4) | 2 (7.4) | 0 (0.0) |
| **MDR bacteria** | 47 (33.1) | 8 (29.6) | 39 (33.9) |
| MRSA | 5 (10.6) | 0 (0.0) | 5 (12.8) |
| VRE | 0 (0.0) | 0 (0.0) | 0 (0.0) |
| 3GC-resistant Enterobacteriaceae (including ESBL) | 30 (63.8) | 6 (75.0) | 24 (61.5) |
| MDR *Pseudomonas aeruginosa* | 1 (2.1) | 0 (0.0) | 1 (2.6) |
| Imipenem-resistant *Acinetobacter* spp. | 4 (8.5) | 0 (0.0) | 4 (10.3) |
| Carbapenem-resistant Enterobacteriaceae | 7 (14.9) | 2 (25.0) | 5 (12.8) |
| **Infection source** |  |  |  |
| Catheter-related BSI | 41 (28.9) | 7 (25.9) | 34 (29.6) |
| Intra-abdominal infection | 4 (2.8) | 1 (3.7) | 3 (2.6) |
| Respiratory tract infection, including HAP, VAP and VAT | 59 (41.5) | 10 (37.0) | 49 (42.6) |
| Urinary tract infection | 3 (2.1) | 1 (3.7) | 2 (1.7) |
| Soft tissue infection | 4 (2.8) | 1 (3.7) | 3 (2.6) |
| Multiple sources of infection | 8 (5.6) | 0 (0.0) | 8 (7.0) |
| Unidentified (primary BSI) | 23 (16.2) | 7 (25.9) | 16 (13.9) |

Values are no. (%) unless otherwise indicated. Rates of specific MDR bacteria calculated among patients with at least one MDR bacteria.

Abbreviations: ICU: intensive care unit; MDR: multidrug-resistant; MRSA: methicillin-resistant Staphylococcus aureus; VRE: vancomycin-resistant enterococci; ESBL: extended spectrum beta-lactamase; 3GC: third-generation cephalosporins.

**Supplementary figure 1. 28-day cumulative incidence of ICU-mortality (a), ICU discharge alive (b) and successful weaning of IMV (c) according to immune status**

A)


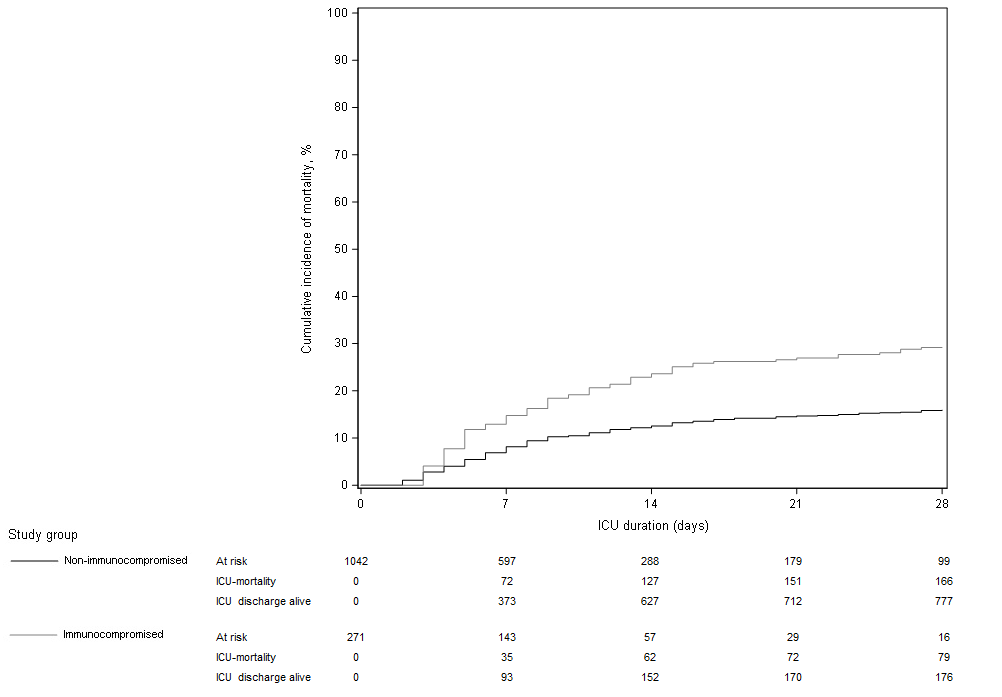


B)


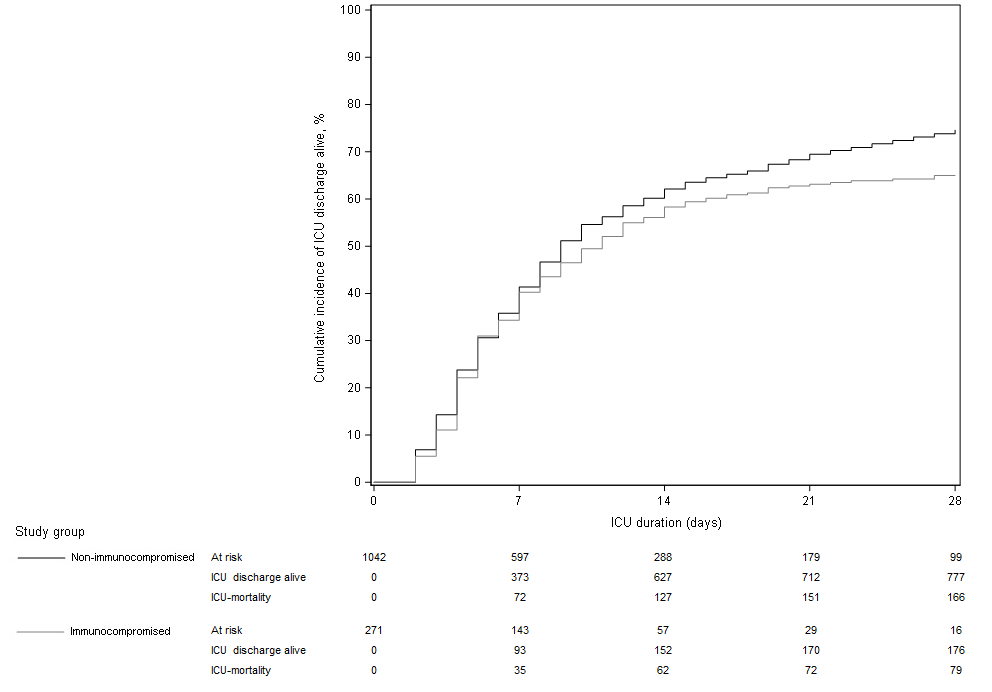


C)


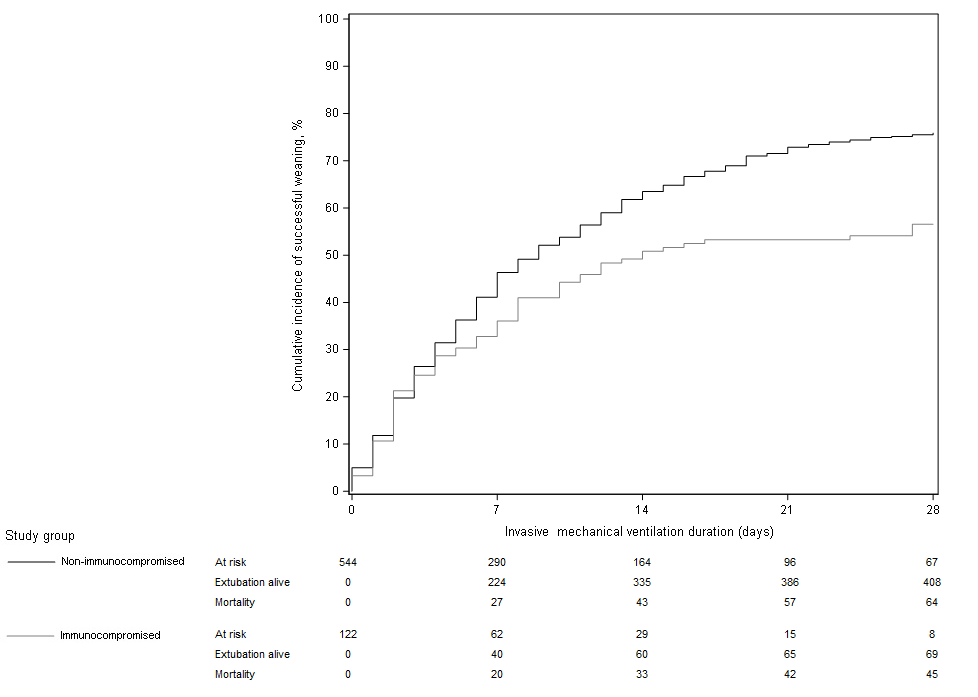

Supplement: Supplementary file 1 — Supplementary Material 1. [file 13613_2024_1314_MOESM1_ESM.docx]
